# Placenta Praevia

**Published:** 1898-03

**Authors:** J. C. Holman

**Affiliations:** Franklin, Texas


For the Texas Medical Journal.

### PLACENTA PRAEVIA.

BY J. C. HOLMAN, M. D., FRANKLIN, TEXAS.

[Read before the Brazos Valley Medical Association.]

Placenta Præevia is a subject of great importance to the medical profession, on account of its dangers, to loss of life, to mother and child; but fortunately it is of rare occurrence, only about one case in one thousand births. Nevertheless, the accoucheur should be familiar with the disease, so as to be able to detect a case, and to utilize the best methods of preventing serious trouble.

It is termed *placenta prævia* when the placenta is attached to the walls of the uterus which are subject to distension during labor. Its clinical importance is proportioned according to the extent of the placental segment which overlaps the *os intenum*. Hence we have to distinguish three varieties:

1st. "*Placenta Prævia Centralis*," where, after the os is dilated, the placenta occludes the os completely, or, in other words, where the placenta is attached around the os internum equally.

2. "*Placenta Prævia Partialis*," or where there is recognizable a portion of the membranes, and also a part of the placenta; or where the attachment of the placenta is not complete around the internal os, and the dilatation of the os brings the margins of the placenta away from the side where there is no attachment, and, on digital examination, the edge of the placenta can be distinctly made out with the membrane.

3d. "*Placenta Prævia Marginalis*," or where the edge of the placenta extends to, but not beyond the inner cervical ring.

The placental attachment never comes below the inner cervical ring, and it is rare to meet with an exact central implantation of the placenta, owing to the deficiency in the thickness of the *decidua* in that vicinity, and the placenta will grow with less profusion at that point, but this is compensated by an excessive development at another point.

You find often the placenta adherent to the uterine walls, the insertion of the cord is often marginal, and as a consequence prolapsus forms a common accompaniment of the anomaly.

THE CAUSES of placenta prævia are not very satisfactorily understood, and there are various theories as to the cause.

The proportion of multiparæ to primiparæ is very large; about six of the former to one of the latter. Statistics show that it is most frequent in women who have borne children with great rapidity, or in pregnancies following immediately after abortions, or such conditions as cause relaxation of the uterine walls.

Some authors claim that the descent of the ovum is brought about by the contractions of the uterus soon after conception, others claim that it is an incomplete abortion, but one most plausible in my mind is that of the relaxed condition of the uterine walls, as when the ovum enters the uterus from the fallopian tubes, if the uterus is in a relaxed state, gravity alone without the contractions of the uterus will cause the ovum to descend, and I think the one greatest cause of placenta prævia is SUBINVOLUTION of the uterus; normally in the uterus of multiparæ, the same as primiparæ, you will find the mucous surfaces nicely coaptated, and nature proposes a reception for the ovum in the enlarged mucous folds close around the opening of the fallopian tubes. *Ingleby* relates two curious cases where the orifices of the fallopian tubes opened near the os internum, in one of which placenta prævia occurred three times, and in the other ten times.

Now as to the cause of placenta prævia in the primiparæ, where the theory of subinvolution could not be plausible, in my mind I do not think that you will meet with a case where the uterus has never been pregnant before. Now don't understand me to say that it could not occur, but I believe it to be so rare that you will not meet with one case in ten thousand cases of placenta prævia. Now conception occurring during menstruation, when the secretions of the womb are very profuse, might become placenta prævia by the flow of the discharge washing the impregnated ovum down to the lower segment of the uterus before it has sufficient time to become adherent to the mucous walls of the uterus.

In cases of placenta prævia in primiparæ, do we know positively that the woman has never conceived. There may be cases of this kind but I rather doubt such.

The clinical features of the greatest importance results from the mode of detachment during labor.

In normal positions the separation of the placenta is effected by virtue of the uterine contractions after the fœtus has for the most part been expelled.

In placenta prævia the separation is due to the stretching, to which the lower uterine zone is subjected in its conversion from a half sphere to a cylindrical canal, to permit the passage of the child. The extent of unavoidable separation in advance of delivery is consequently measured by the dimensions of the child's head, the largest circumference of which is estimated as equivelent to a circle with a diameter of four and one-half inches. According to Duncan the plane at which spontaneous detachment ceases is reached at a distance of two and one-half inches by following the curve of the lower segment, and of one and one-half inches if measured in the direction of the uterine axis, whereas in normal labor the contractions of the uterus which determine placental separation cease at the same time the orifices of the torn vessels close.

The stretching of the lower segment of the uterus in placenta prævia leaves the mouths of the sinuses open, from which the blood pours until the stream is arrested either by art or by the supervention of syncope. The hemorrhage of placenta prævia is not, however, limited to the parturient period. There is no time during pregnancy in which it may not occur, as every jar or jolt of the body effects the lower segment of the uterus with more force than it does the fundus, and the thinned walls of the utero placental vessels are subject to greater pressure in placenta prævia. So you may expect hemorrhage from the slightest misstep. Thus you may expect pseudo menstruation of pregnancy, a predisposition to abortion, and later a premature labor; although you may find cases that go to full term without the least hemorrhage.

"PROGNOSIS" in cases of placenta prævia is very unfavorable. As many as one mother out of four dies during or shortly after delivery. Then after the uterus has been emptied of its contents and firmly contracted it often relaxes suddenly and the blood gushes forth in torrents. Then we have the dangers of sepsis, as the clots that fill the sinuses are very apt to become infected and the decomposition cause pyæmia or septicæmia.

Muller says from thirty-six to forty per cent of all cases die. Nearly two out of three of the children are born dead. More than one-half of those born living die within the first ten days.

The earlier in pregnancy the hemorrhage appears, the more serious is the prognosis, the more profuse the flow, the shorter the intervals between attacks.

Favorable conditions during labor are vertex presentation, good pains, rapid dilatation, and a good constitution.

Placenta prævia centralis seems to cause greater mortality than either of the other two forms, double that of placenta prævia lateralis.

In the crowded city there is the special danger of infection, in the country the delay in securing medical assistance, and after all it is hard to estimate the dangers correctly as to results without coming to the conclusion that the results depend to a great extent on the quality of the medical attendant. A self-possessed man, cool, resolute, with clear ideas as to the anatomical condition to be dealt with will, if summoned in due time, deprive placenta prævia of a good share of its terrors.

The diagnosis is very indistinct and unsatisfactory in the first half of pregnancy and I think nearly an impossibility.

It may occasion abortion which is then characterized by the absence of pain both previous to hemorrhage and during the period of expulsion; generally the ovum is expelled in mass without rupture of the membranes. During the latter part of pregnancy a sudden gush of blood without perceptible cause and without warning should always be regarded with suspicion.

Upon digital examination in placenta prævia the vaginal fornix will be found soft and boggy and occasionally more bulky on one side than the other, i. e,, where placental presentation is incomplete.

Ballottement can hardly ever be practiced with any satisfaction; the cervix is long, wide and soft, and often the pulsation of the blood vessels can be distinctly felt; the cervix admits the finger to the internal os which offers resistance but yields to a slight force. Now the only actual and positively accurate diagnostic symptom is the presence of the placenta to be felt with the finger through the cervix. Its rough, spongy, granular feel is sufficient to distinguish it from clots and other sources of deception.

In the treatment of placenta prævia, the history brings into prominence the main point to be kept in view constantly in the practice, that there is no safety for the mother so long as pregnancy continues. In the majority of cases accidental hemorrhage occurring in the first half of pregnancy leads to abortion, however, the management of this does not differ from that of normal attachments of the placenta, and the mortality caused by placenta prævia prior to the seventh month is very low; however, there is an occasional death during the first seven months of gestation, but due to a lack of knowledge on the part of the attendant, death resulting generally from loss of blood or septic infection.

In the latter half of pregnancy hemorrhage leads to premature expulsion of the ovum with such frequency that it is reckoned that only about one-half of all cases reach the full term of gestation.

The hemorrhages of placenta prævia are usually sudden, with no warning, no pain, often without any apparent cause; sometimes occurring at the time of urination, sometimes during sleep; often as the woman walks across the floor a clot will escape.

The hemorrhage may be very little and may not occur often, the amount of blood lost varies, depends upon the extent of the placental separation; the first outpouring may lead to intense anæmia and if repeated may cause death.

(As a rule the hemorrhages of pregnancy are at first moderate in character, increasing in violence with each successive pregnancy.)

A very formidable variety is the so-called Stillicidium, where the blood issues drop by drop for days and even weeks in succession.

But the most violent hemorrhages occur generally in the earlier part of the first stage of labor, and the amount of hemorrhage depends on the area of the placental attachment occupying that portion of the uterus subject to distention. The hemorrhage generally ceases after the membranes have ruptured and the presenting part is brought to bear upon the bleeding surface; and during the height of pain the blood is momentarily checked but will begin again as soon as the womb is relaxed.

There is a great number of abnormal presentations in placenta prævia caused, I think, by the large proportion of premature labors, and partly by the condition of the lower segment of the uterus and the consequent stability of the fœtus.

During the first stage of labor the pains are apt to be feeble, and the dilatation tardy. The cause of this inertia must be in the thinning of the muscular structures in the lower segment by the enormous development of the utero placental vessels in the placenta over the os, which mechanically hinders dilatation, and the nerves are not stimulated by the direct pressure on the cervix.

Secondary weakness often follows the continued loss of blood, and the prolongation of the first stage, when the obstacle to dilatation afforded by the placenta is overcome, and following the rupture of the membranes, the uterus retracts; and in many instances the scene speedily changes, and in place of ineffective contractions, normal and often powerful labor pains develop, and delivery immediately follows.

Hemorrhage occurring earlier than the seventh month, or prior to the time of viability, I think the chances too small for the child, to try to carry the woman to full term, hence immediate delivery, as the wisdom of delay is open to serious question.

The fatality of placenta prævia is due not so much to the impotence of obstetrical art as to the losses of blood, which occur suddenly in the absence of professional assistance. The first hemorrhage, which serves as a warning of the patient's condition, is fortunately in most cases slight but with each recurrence it becomes more profuse. Now, to trifle with such cases is the best way to maintain the present mournful statistics.

After the thirty-second week it is safe to say that the child's life is less imperiled by the induction of premature labor than by exposing it to the dangers of continued gestation. Then, on theoretical grounds, the induction of premature labor is to be regarded as obligatory so soon as the diagnosis of placenta prævia is established, or at least with the appearance of the first hemorrhage.

If the pregnancy has advanced beyond the seventh month it will as a rule, I think, be wise to proceed at once to delivery, for the next hemorrhage may be fatal. We cannot tell the time or extent of its occurrence and when it occurs perhaps all that we will have the opportunity to do will be to regret that we did not act when we had the chance.

In the management of placenta prævia it is very desirable that the practitioner should have a perfectly clear idea of the nature of the task he has to perform. The birth of the child cannot take place without preliminary expansion of the cervix; the cervix cannot expand without detachment of the placenta. Now, the principle point of treatment is the hemorrhage which occurs during the stage of dilatation.

Plans without number for restricting the flow within narrow limits have been proposed by the masters of the obstetrical art. The best plans are those which at the same time contribute to shorten labor; the choice must be determined by conditions which necessarily vary in different cases.

The physician has at the outset to inform himself as to whether labor has begun or remains to be inaugurated; as to whether the placenta prævia is complete or incomplete; as to whether the presentation is normal and the pains good; as to the length and dilatation of the cervix. If the cervix is long, narrow and rigid, the vaginal tampon should be used as a temporary expedient. The tampon strengthens the pains, causes the blood to clot, and reduces the loss of blood. Thorough asepsis should be regarded as essential here as in a case of abdominal section.

The tampons can be made of cotton made into discs just large enough to be passed through a Sims speculum, these discs to be dampened with a two per cent. solution of carbolic acid and glycerine, then to be well crowded up in the vagina until it is well packed. Having the vagina tamponed the physician should not leave his patient until the labor is ended. After four to six hours the tampons should be removed, and in almost every case you will find the cervix somewhat dilated or dilatable. However, if it is not dilated any more than to admit the finger then you should wash out the vagina with antiseptic solution and proceed to dilate with the hot water bag (Barnes' rubber bag), sufficiently to admit the hand enough to grasp the feet and bring the breech down, and then you can let nature take her time, as the breech acts as a plug and checks all hemorrhage.

Now, if the placenta is so attached that this cannot be done take a fountain syringe filled with very hot water and with the water flowing separate the placenta from the uterus with the nozzle of the syringe; the hot water acts as a styptic and an antiseptic. Now, when the separation is complete, just pass the hand on into the uterus and bring the breech down, then you can take your time.

Some practitioners prefer sponge tents to dilate the os with, but on account of their being so hard to render aseptic, I prefer the rubber bag.

If no urgent symptoms call for immediate interference, it is desirable that the dilatation should be as near complete as possible.

In lateral attachments of the placenta after the os has been dilated and the presenting part engaged, whether breech or head, and acts as a plug to check the hemorrhage I would rather let nature take its course.

But unfortunately the physician does not have this opportunity every time. He is called hastily to a woman in labor; is told that she is flooding to death. He enters the room and finds her in a dying condition, face blanched and covered with a cold clammy sweat, gasping for breath, pulse hardly perceptible, and the woman in a pool of her own blood. Now, this is where the physician should be cool, and proceed to act promptly. There are two objects now in view; first, to revive his patient; second, to check the hemorrhage. Stimulants, such as brandy, aromatic spirits of ammonia, strychnia, all in heroic doses, should be given. The feet of the patient should be elevated by placing bricks or blocks under the bed posts; let the head down by removing the pillows; apply external warmth, as hot water bottles and warm blankets. In a case of this kind I would surely give ergot in heroic doses, but would proceed to deliver at once for the child would be dead in a very short while.

Now, in a case like this, where the os is not dilated sufficiently to do version, then I would resort to the tampon.

A case of this sort should never be left, though the hemorrhage is checked, the patient revived and in good condition, the physician should never leave her until the uterus is emptied of its contents and contracted firmly, as such cases will undoubtedly relapse into the same condition sooner or later and you may not be fortunate enough again to be in time to save life. And especially in such cases as this should aseptic precautions be carried out, both during and after delivery. After delivery the vagina should be douched twice daily for several days, with a strong bichloride solution, say 1 in 1000, or carbolic solution, 2 or 3 per cent., as the lochial discharge, passing over the placental wound is so very apt to cause infection and serious trouble if not death.

The result of delay in cases of placenta prævia will be made more impressive by relating my experience with a case, and how near death came to my patient's door by my not acting properly when I had the opportunity.

Was called to see a colored woman on the night of Oct. 7th; her husband told me she was about to abort. When I arrived at the bedside I found her to be about 22 years old, the mother of a boy about nine months old, and found her to be about five months advanced in pregnancy. The first she noticed anything wrong was some two weeks ago, this being slight blood stains on her linen. On the evening before I was called that night, she jumped from a cotton wagon and some hours afterwards several clots passed and the alarm was caused by the continued oozing which was increasing instead of diminishing. I at once suspected placenta prævia as she had not had any pains at all or any symptoms of abortion, only the hemorrhage.

I prepared myself and made a digital examination; found the cervix low down, enlarged and soft; just the least dilatation of the os but not sufficient to admit the finger. I now proceeded to tampon the vagina. This being done I let her rest about five hours then removed the tampons and made a second examination; found the cervix firmly contracted, the hemorrhage had ceased and the patient was resting well.

I prescribed acid drinks, acetate of lead and tincture of opium, and rest in bed; told the husband to report to me if the hemorrhage recurred, and left. On the morning of the 9th at 4

o'clock her husband came and said she was flooding to death. I hastened to the scene, found her gasping for breath, pulse almost gone, cold sweat on face, the blood dripping on the floor through the bedclothes, she in a pool of blood. I lowered her head, raised her feet, gave a stimulant of strychnia, spirits ammonia arom. with the needle, and made a hasty examination. Found cervix soft, os dilated size of 25-cent piece, and when the fingers entered the cervix to the inner os the placenta was felt to be attached evenly around the inner os, the blood was still flowing profusely. I used the hot water douche, the water so hot that it would almost scald my hand, passing the nozzle around the attachment of the placenta. This checked the hemorrhage but the os was not dilated sufficiently to get the hand in far enough to peel the placenta off. I then tamponed the vagina well and sent for assistance. When he arrived I gave a good stimulant, removed the tampon and gave chloroform and introduced the hand, peeled off the placental attachment, brought the fœtus into the os, held it there, gave thirty drops of ergotole with the needle, and let the contractions of the womb expel the fœtus and secundines. I then douched the womb with a very hot carbolic solution. There was but very little after-hemorrhage, no more than from a normal labor, if as much. The womb contracted down finely and the patient was soon resting well, and seemed to suffer no shock whatever.

On the 2nd day the temperature rose; I washed out the womb with hot water and filled it with campho-phenique, as that was all that I had that would answer the place of carbolic acid. She made a good recovery. The fœtus proved to be five months old, conception taking place about four months after delivery of the first born.

